# Molecular docking analysis of azithromycin and hydroxychloroquine with spike surface glycoprotein of SARS-CoV-2

**DOI:** 10.6026/97320630017011

**Published:** 2021-01-31

**Authors:** Jitender Singh, Deepti Malik, Ashvinder Raina

**Affiliations:** 1 Post Graduate Institute of Medical Education and Research, Sector-12, Chandigarh, India. Pin-160012

**Keywords:** Drugs, pharmacokinetics, binding pocket, molecular docking, structure activity relationship

## Abstract

Millions of people are affected by COVID-19 since the last quarter of 2019. Treatment using hydroxychloroquine (HCQ) as monotherapy in combination with azithromycin (HCQ-AZ) were administered at several clinical centres to patients tested positive to the virus across continents. Therefore, it is of interest to document the molecular docking analysis data of azithromycin and hydroxychloroquine drug with the spike surface glycoprotein of novel COVID-19. Thus, we report the molecular modelling docking based structural binding features of HCQ-AZ with the spike surface glycoprotein of COVID-19 for further evaluation in this regard.

## Background

COVID-19 in humans has emerged in Wuhan, China. Its genome has been sequenced and the genomic information promptly released. Despite a high similarity with the genome sequence of SARS-CoV and SARS-like CoVs, we identified a peculiar furin-like cleavage site in the Spike protein of the 2019-nCoV, lacking in the other SARS-like CoVs [1]. In this research article, we discuss the possible functional impact of HCQ-AZ on the spike protein, pathogenicity and its potential implication in the development of COVID-19 therapy. Human coronaviruses (CoV) are enveloped positive-stranded RNA viruses belonging to the order Nido Virales and are mostly responsible for upper respiratory and digestive tract infections. Among them SARS-CoV and MERS-CoV that spread in 2002 and 2013 respectively, have been associated with severe human illnesses, such as severe pneumonia and bronchiolitis, and even meningitis in more vulnerable populations [2]. In December 2019, a new CoV (2019-nCoV) has been detected in the city of Wuhan, and this emerging viral infection was associated with severe human respiratory disease with a ∼2-3% fatality rate [3]. In this article, we focus on a specific furin-like protease recognition pattern present in the vicinity of one of the maturation sites of the S protein and the impact of HCQ-AZ inhibiting this spike. Coronavirus spike (S) glycoproteins promote entry into cells and are the main target of antibodies. SARS-CoV-2 S uses ACE2 to enter cells and that the receptor-binding domains of SARS-CoV-2 S and SARS-CoV S bind with similar affinities to human ACE2, correlating with the efficient spread of SARS-CoV-2 among humans [4]. The coronavirus spike (S) glycoprotein initiates infection by promoting fusion of the viral and cellular membranes through conformational changes that remains largely uncharacterized. Coronavirus entry is mediated by the trimeric transmembrane spike (S) glycoprotein, which is responsible for receptor binding and fusion of the viral and host membranes. S is a class I viral fusion protein that is synthesized as a single-chain precursor of -1, 300 amino acids and trimerizes upon folding. It forms an extensive crown decorating the virus surface and is the main target of neutralizing antibodies upon infection [5]. Therefore, it is of interest to document the molecular docking analysis data of azithromycin and hydroxychloroquine drug with the spike surface glycoprotein of novel COVID-19.

## Methodology

### Protein preparation:

Primary sequence of surface glycoprotein of severe acute respiratory syndrome coronavirus 2 with protein NCBI ID: YP_009724390.1 was retrieved in FASTA format from National Centre for Biotechnology Information (http://www.ncbi.nlm.nih.gov/) database which has been recently updated in NCBI database. The 3D structure of surface glycoprotein was constructed using SWISS MODEL server (https://swissmodel.expasy.org/). The 3D model of the target protein generated by us was also available in the Swiss model database. The best model was selected from the Swiss model database. Plotting the Ramachandran plot did further validation of the 3D structure model and MolProbity Score1.50 was taken from SWISS MODEL database.

### Binding site prediction:

Protein-Ligand-binding sites are the active sites on protein surface that perform protein functions. Thus, the identification of these binding sites is often the first step to study protein functions and structure-based drug design. Active sites (ligand binding sites) of the receptor protein were analysed by Metapocket 2.0 online tool (https://projects.biotec.tu-dresden.de/metapocket/). Binding sites are the distribution of surrounding residues in the active sites and act as the catalytic residues [6].

### Antigenic peptides prediction:

Antigenicity is the capacity of an antigen to bind specifically with a group of certain products that have adaptive immunity: T cell receptors or antibodies. Antigenic determinants were analysed using ImmunoMedicine Group Server Antigenic Peptide Prediction (http://imed.med.ucm.es/Tools/index.html).

### Ligand preparation:

The ligands azithromycin and hydroxychloroquine were selected for binding with the target protein (spike surface glycoprotein) of COVID-19. The structure of these drug compounds was downloaded from PubChem database (http://pubchem.ncbi.nlm.nih.gov/). The drug files was downloaded in SDF file format and were converted into PDB files using open PyMOL software [7] (and Discovery Visualize Software [8]. The ligands preparation included 2D-3D conversion, verifying and optimizing the structures. We have taken two FDA approved drug molecule structures for docking analysis with target surface glycoprotein.

### Docking:

AutoDock vina (PyRx) software was used to dock protein and ligand molecules. Molecular docking of selected drug molecules at the binding site of target protein of COVID-19 was docked by automated docking program AutoDock vina (PyRx) [9]. Preparation of required input files for docking was completed using AutoDock Vina (PyRx) bioinformatics software. The target receptor file and all ligand files were uploaded to the software for the docking with individual drug molecules. An autogrid map dimensions was set to find the best binding site in the coordinating centre. The results were analysed after the docking process was complete.

## Results and Discussion:

**Figure 1:**
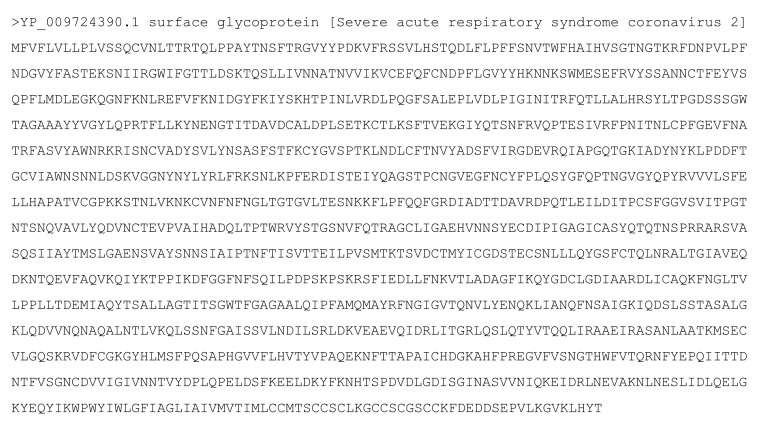
Analysis of Primary sequence characterization of spike surface glycoprotein of COVID-19: Snapshot of 1273 amino acid single chain long primary sequence of spike surface protein of COVID-19 retrieved from NCBI database having NCBI ID: YP_009724390.1

**Figure 2:**
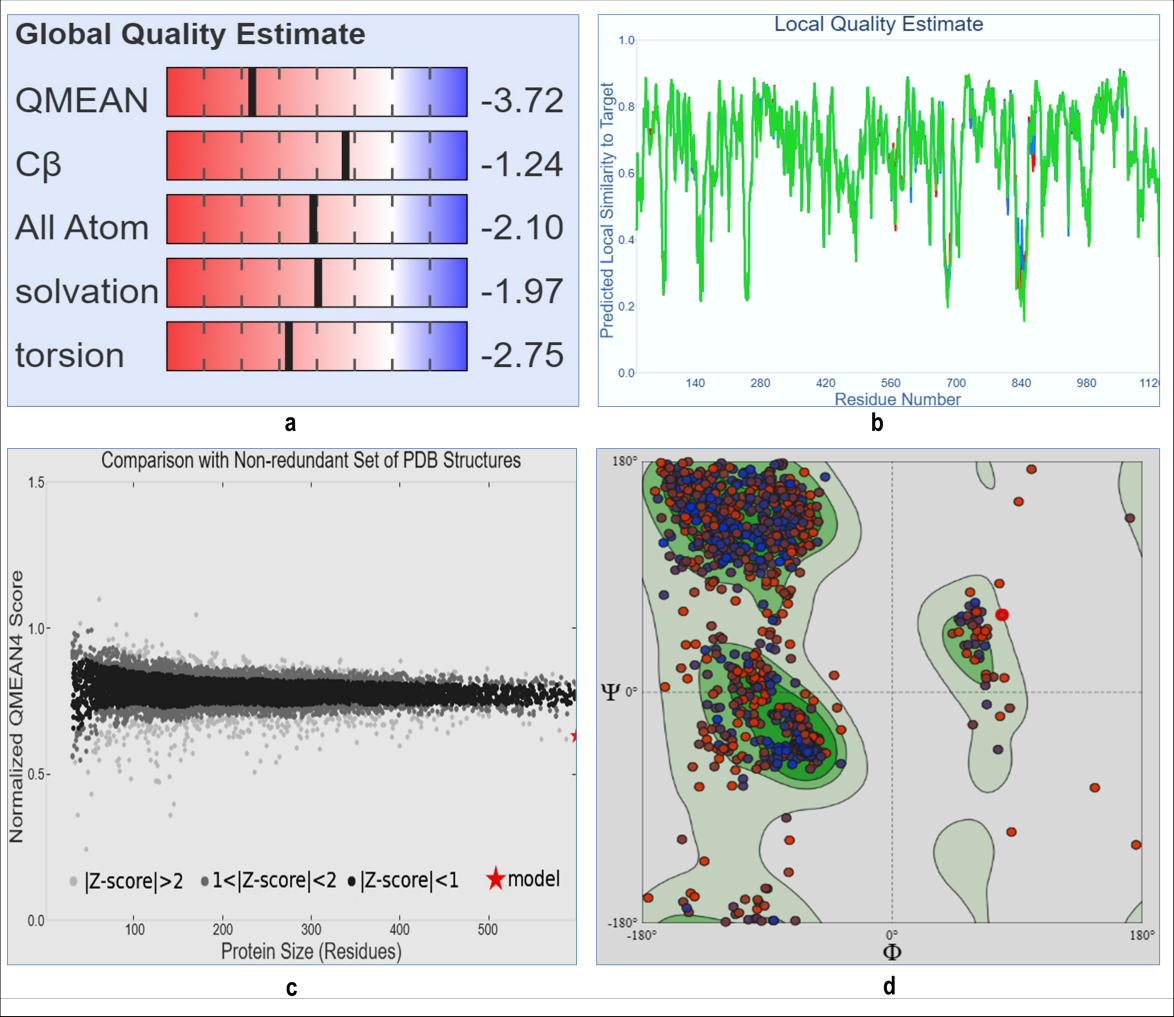
Model quality and Ramachandran plot of spike surface glycoprotein of COVID-19: a) Showing global quality estimation of target protein which has -3.72 QMEAN value. b) Local quality estimation of S protein of COVID-19, having maximum number of residues showing scores more than 0.8. C) z-score of modelled structure which comparison with non-redundant set of PDB structure. d) Ramachandran plot retrieved from SWISS-MODEL database of target protein representing 97.50% of the favoured residues in the plot area with good validate score thus can be considered as a target molecule.

**Figure 3:**
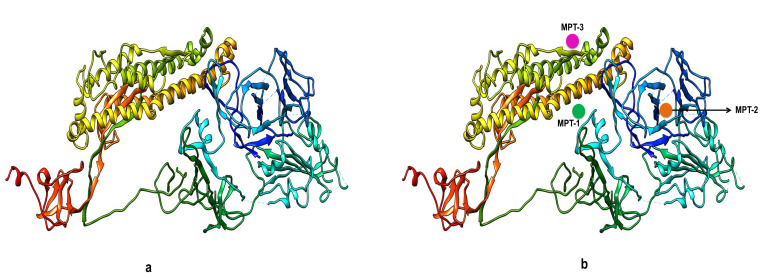
Functional and structural characterization of spike surface glycoprotein of COVID-19: a) Representative 3D modelled structure of target protein (spike surface glycoprotein) generated through SWISS-MODEL database. b) Representation of top three metaPocket regions within 3D carton structure marked as metaPocket-1, 2, 3 with good pocket z-score: 20.28 of 3 pocket sites. The amino acid positions within these meta-Pockets were analysed using Metapocket2.0 online tool (https://projects.biotec.tu-dresden. de/metapocket/).

**Figure 4:**
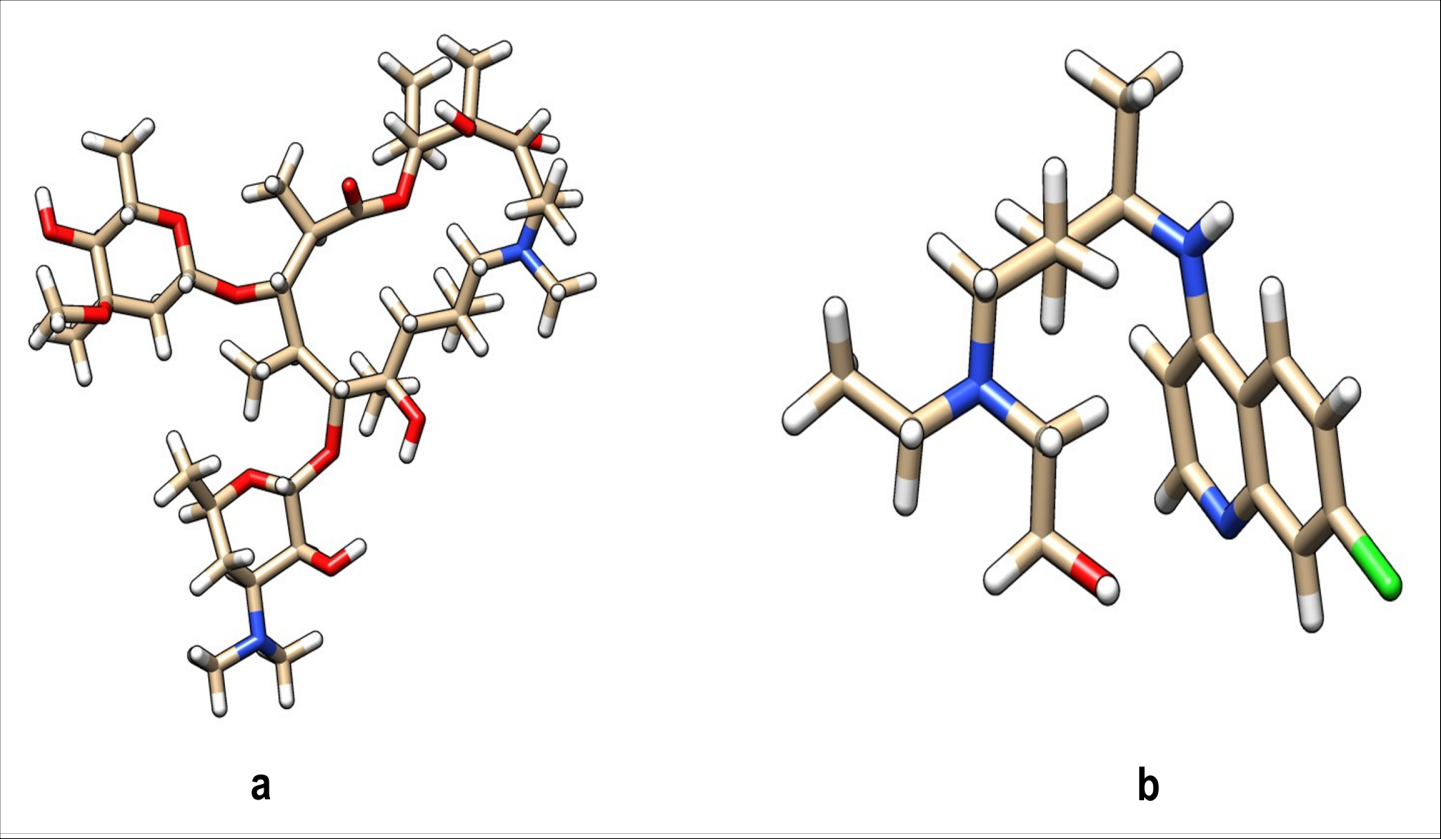
Structural and chemical properties of selected drug molecules: Representative 3D structure of a) azithromycin b) hydroxychloroquine. SDF structure retrieved from PUBCHEM database was converted to PDB structure using Discovery Studio Visualizer free software.

**Figure 5:**
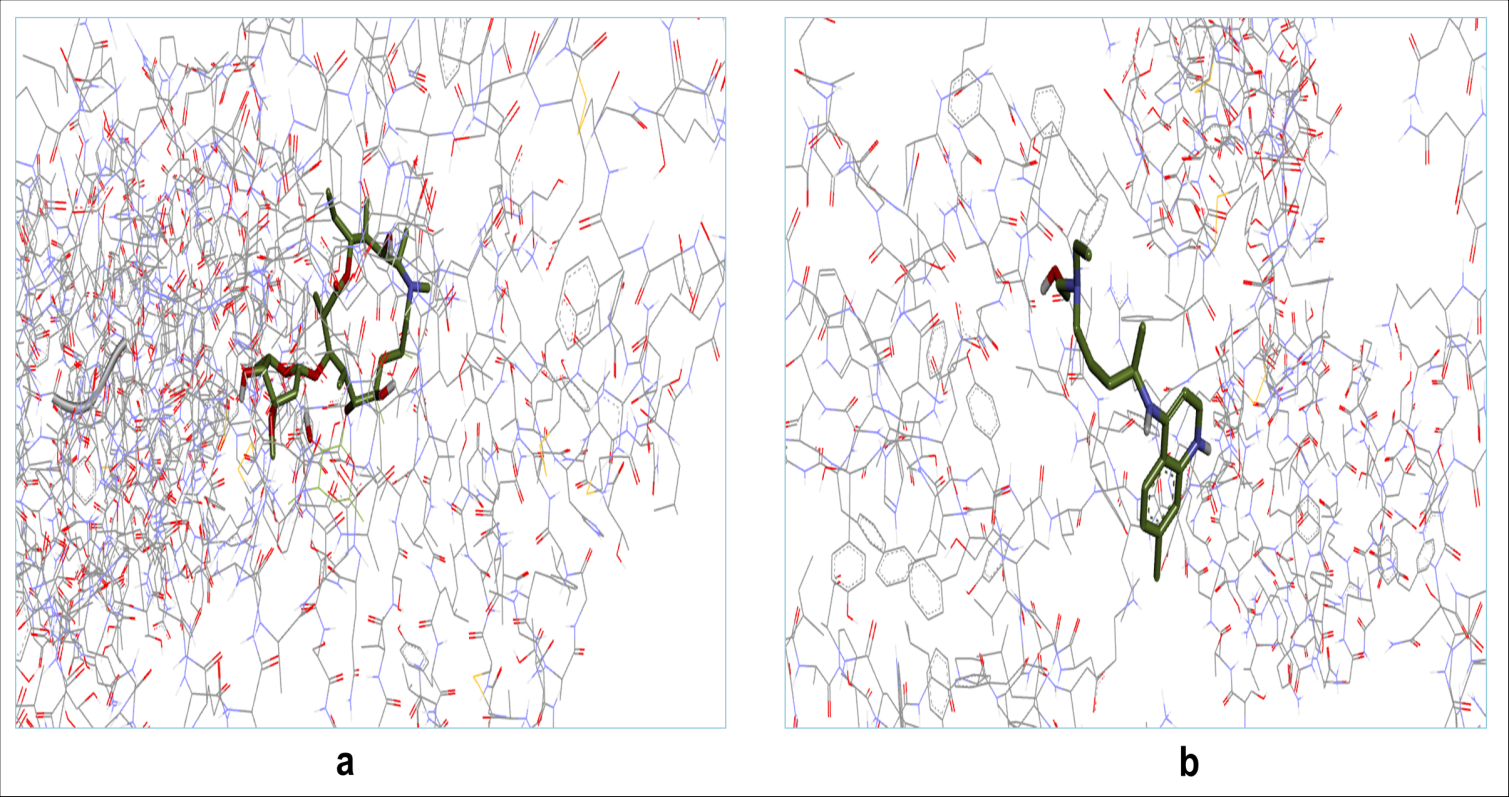
Molecular Modeling and docking studies of both selected drugs with surface glycoprotein of COVID-19: Representative 3D structure of complex docked target protein molecule (surface glycoprotein) with a) azithromycin, b) hydroxychloroquine.

**Figure 6:**
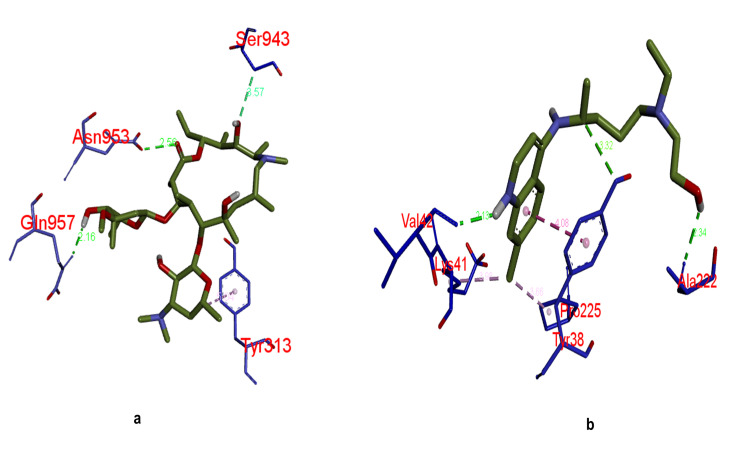
3D docking binding interactions of both selected drugs with surface glycoprotein of COVID-19. Bonding interaction(s) between drug molecules and target protein receptor; (a) target protein + azithromycin; (b) target protein + hydroxychloroquine.

**Figure 7:**
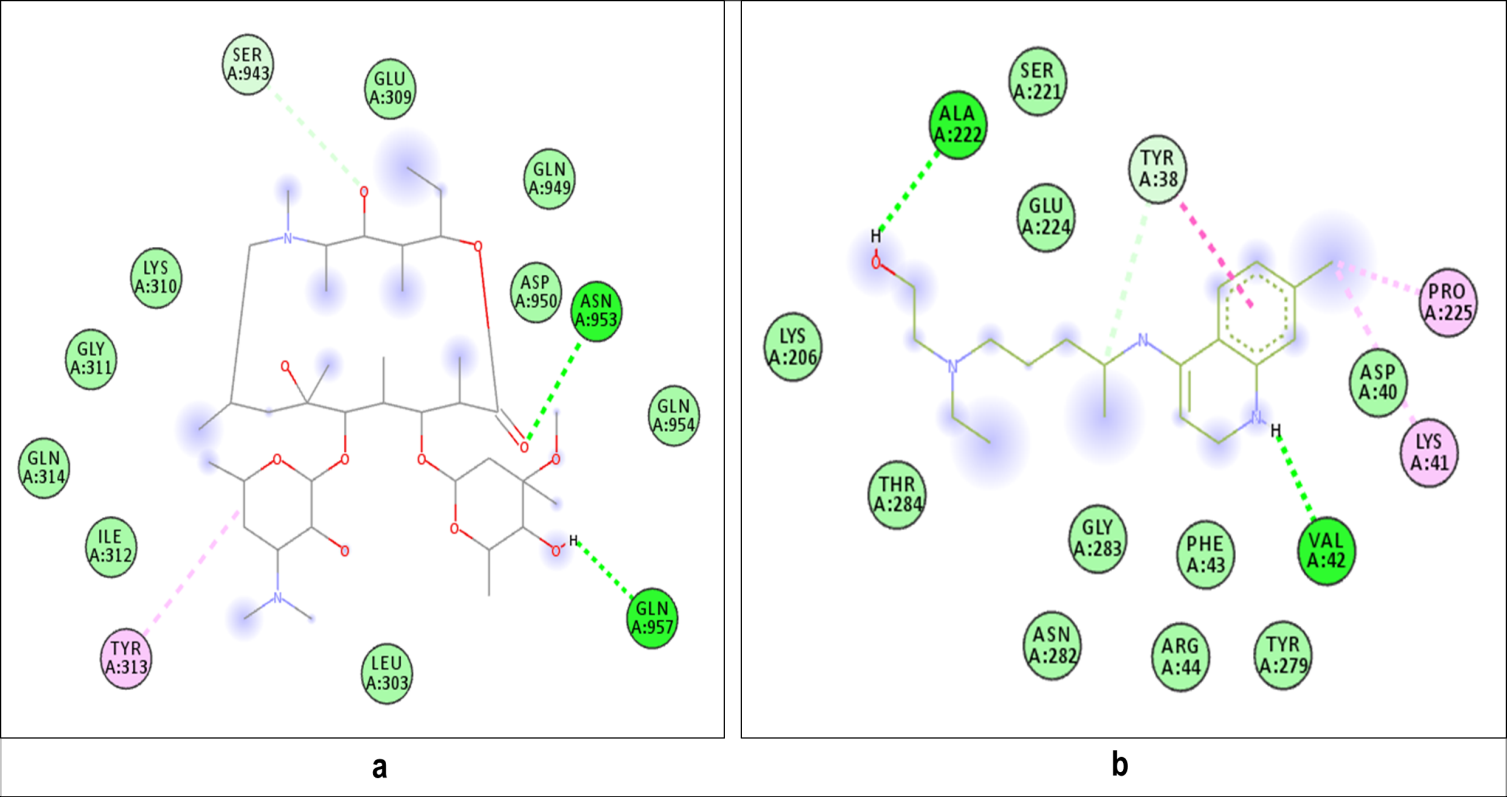
Representation of 2D complex structure of both drugs and target protein indicating different types of bonds interacting with various amino acid residues: a) Target protein + azithromycin. b) Target protein + hydroxychloroquine . All the 2D structures were generated from the Discovery Studio Visualize software and PYMOL Visualize.

**Figure 8:**
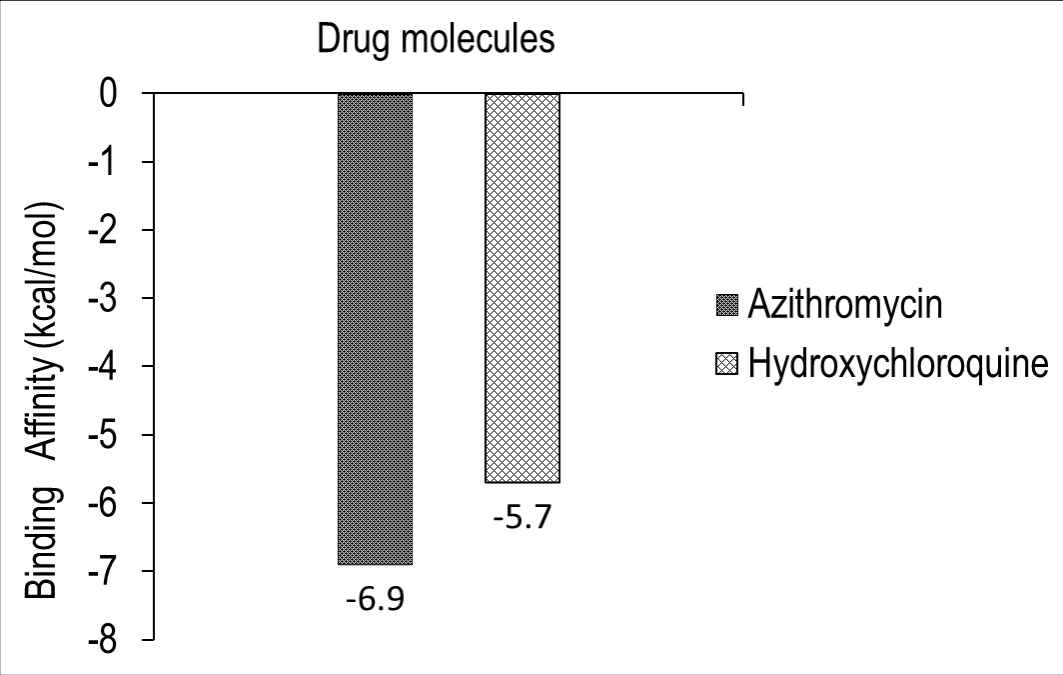
Graphical representation of binding energy of both the drugs with surface target glycoprotein from minimum to maximum energy score in kcal/mol.

The 1273 amino acids residue long primary sequence of spike surface glycoprotein of severe acute respiratory syndrome coronavirus 2 with protein NCBI ID: YP_009724390.1 was retrieved in FASTA format from National Centre for Biotechnology Information database (Figure 1). 3D structures of target protein spike surface glycoprotein of COVID-19 were built with the help of SWISS MODEL server. The modelled structure is also available on the SWISS MODEL database. The best model was selected from the SWISS MODEL database which was having 99.26% sequence identity with the template sequence of PDB ID: 6VSB. After the generation of 3D structure of target protein, the model quality was checked with SWISS MODEL online program. The SWISS MODEL online program showed that the "target" protein model, which was generated, was the best model structure, with good global quality and having best Z score for quality of the structures. The GMQE value was 0.73 and QMEAN score was -3.72 (Figure 2a-c). For the prediction of the active binding site(s) of "target" protein structure, first we validated the 3D structure of target" protein using Ramachandran plot and we found 90.49% residues in the favoured region (Figure 2d). The final predicted 3D structure model of the target proteins is as shown in (Figure 3a). After validation of this predicted 3D structure of target protein, next we wanted to explore ligand binding site residues in surface glycoprotein using Meta Pocket 2.0 Web online tool. On analysis the 3D structure of target protein of COVID-19, we found there were top three active sites (meta-pockets), considered as ligand binding sites in this protein (Figure 3b). Analysing the number of important amino acid residues in the catalytic region followed site prediction. Important amino acid residues present in these top three metaPocket sites are shown in Table 1(see PDF). These three metaPocket(s) were studied for further analysis as they had important amino acid residues. Next we predicted the antigenicity of target surface glycoprotein using online server Immuno Medicine group tool Antigenic Peptide Prediction. On analysis, we found there were 63 antigenic determinants found in the target sequence of surface glycoprotein with average antigenic propensity score is 1.0416 which is considered to be a valid score as shown in Table 2 (see PDF). After the confirmation of Meta Pocket site(s) and antigenic peptide prediction, two drug compounds were selected as ligands for docking with the selected spike surface glycoprotein of COVID-19 protein. 3D structure of both the chemical compounds was downloaded from the NCBI PubChem database (Figure 4a-b). These two compounds azithromycin and hydroxychloroquine, which are already FDA, established drugs and therefore follow the Lipinski rule of five to evaluate drug-likeness (www.scfbio-iitd.res.in) [10]. These two drug compounds were then checked for their binding with target protein and docking score was evaluated using AutoDock vina Docking (PyRx) software. An autogrid map dimension was set at grid centre X: 2000.63, Y: 227.356, Z: 217.7743. Number of point was X: 68, Y: 58, Z: 76 and spacing was 0.9873 Angstrom. For each compound, many docking poses were obtained for target protein with different binding affinity score. Out of many docking poses, only those were selected which had the best and highest docking score, good hydrogen bond interaction and highest binding energy. The binding interaction(s) of all the docked with complex structure of selected two drug molecules are shown in Figure 5a-b. The 3D interactions of receptor target protein of COVID-19 with these two selected drugs are represented in Figure 6a-b. Similarly the 2D poses of receptor target protein of COVID-19 with selected two drugs are represented in Figure 7a-b. Different types of bonding interactions were observed between the same and it is a universal acknowledged fact that hydrogen bonding is of considerable importance in the interaction between such molecules and from our results we also found that both the selected drug molecules showed highest binding energy with the target surface glycoprotein of COVID-19 (Table 3 - see PDF). We observed that the binding affinity score of Azithromycin is -6.9kcal/mol and that of hydroxychloroquine is -5.7 kcal/mol, which is considered as good docking score. Graphical representation of the same is shown in Figure 8. In addition to this both complex structure showed 3 Hydrogen bond interactions (Table 3 - see PDF). In coherence to our study reported the binding energies obtained from the docking of S protein with ligand, chloroquine and hydroxychloroquine were -7.1 and -6.8kcal/mol [11]. Another study reported the binding affinity of phytocompounds derived from Silybum marianum (Silybin), Withania somnifera (Withaferin A), Tinospora cordifolia (Cordioside) and Aloe barbadensis (Catechin and Quercetin) with SARS-CoV-2 target [12]. Other studies are also there which underline the significance of Hydroxychloroquine and Azithromycin in COVID-19 [13-17]. From these findings, the study concluded that these two selected drug molecules azithromycin and hydroxychloroquine may act as potential candidate drug molecules against the target spike surface glycoprotein of COVID-19.

## Conclusion

The focus of our study was molecular docking of azithromycin and hydroxychloroquine drug compounds, which potentially have therapeutic impact on spike surface glycoprotein of novel COVID-19. Docked result confirmed our hypothesis that both the selected drug molecules bind to surface glycoprotein protein of COVID-19 showing best binding affinity which attests to the good binding of these drugs to the target protein. On the basis of molecular docking energy score of both selected FDA approved drug molecules suggest that they may act as inhibitor that can potentially be used for the treatment of severe acute respiratory syndrome coronavirus 2. Based on results we report the molecular modelling docking based structural binding features of HCQ-AZ with the spike surface glycoprotein of COVID-19 for further evaluation in this regard.

## References

[1] Coutar B (2020). Antiviral Res..

[2] De Wit E (2016). Nat Rev Microbiol..

[3] She J (2020). Clin Transl Med..

[4] Walls AC (2020). Cell.

[5] Walls AC (2017). Proc Natl Acad Sci U S A..

[6] Zhang Z (2011). Bioinformatics.

[7] https://pymol.org/2/.

[8] https://www.3ds.com/.

[9] Trott O, Olson AJ (2010). Journal of Computational Chemistry..

[10] Lipinski CA (2004). Drug Discov Today Technol..

[11] Seshu Vardhan (2020). Comput Biol Med.

[12] Nabajyoti Baildya (2020). Mol Struct..

[13] Gautret P (2020). Int J Antimicrob Agents..

[14] Choudhary R, Sharma AK (2020). New Microbes New Infect..

[15] Arshad S (2020). Int J Infect Dis..

[16] Rosenberg ES (2020). JAMA..

[17] Gautret P (2020). Travel Med Infect Dis..

[18] http://www.ncbi.nlm.nih.gov/.

[19] https://swissmodel.expasy.org/.

[20] https://projects.biotec.tu-dresden.de/metapocket/.

[21] http://imed.med.ucm.es/Tools/index.html.

[22] http://www.scfbio-iitd.res.in.

